# Environmental and occupational respiratory diseases – 1033. Rust fungi of plants and high IgE levels in asymptomatic workers of a stored food grains godown: a possible relationship

**DOI:** 10.1186/1939-4551-6-S1-P32

**Published:** 2013-04-23

**Authors:** Satadal Das, Debkishore Gupta

**Affiliations:** 1 Department of Microbiology, Peerless Hospital & B. K. Roy Research Centre, Kolkata, India

## Background

Rust fungi are often isolated in air samples collected from storage areas, however,their role in allergy is not investigated so far. Thus this study was designed to find out anypossible role of rust fungi in human allergy.

## Methods

Air samples were collected during loading/unloading of stored food grains in a godown with the help of Rotorod sampler, UK on petroleum jelly coated tape at 2900 rpm for 30 minutes, the tapes were then directly inoculated on Sabouraud’s dextrose media for isolation of fungi by imprint smear. Most of the collected samples yield *Aspergillus* sp., *Rhizopus* sp., *Penicillium* sp., *Mucor* sp. and along with them one variety of Rust fungi of plants was also repeatedly isolated. Formaldehyde based fungal suspensions were prepared from all the cultured fungi, workers' blood samples were collected and serums were separated. Then serum samples were teated with the formalin suspensions of the isolated fungi in the form of slide agglutination tests. Serum total IgE levels of all collected blood samples were also measured by ELISA test method.

## Results

It was found that the isolated Rust fungus of plants gave positive agglutination tests with almost all collected serum sampleswhile agglutination reactions with the other isolated fungi were much less. The reactions of the isolated Rust fungi were violent with serums showing high IgE levels. Serum IgE levels were also very high in many of the workers.

## Conclusions

This study indicates that Rust fungi of plants have got a role in human allergy and details regarding this should be explored for the benefit of the mankind.

